# Rare Intramuscular Myxoma Involving the Pterygoid and Masseteric Muscles: A Case Report

**DOI:** 10.7759/cureus.49772

**Published:** 2023-12-01

**Authors:** Faisal S Alharthi, Khaled A Alasmari, Sultan H Alruwaili, Suhail M Basuhail, Tareq A Hamad

**Affiliations:** 1 Otolaryngology, King Fahad Specialist Hospital, Tabuk, SAU; 2 General Practice, University of Tabuk, Tabuk, SAU; 3 Otolaryngology, Eastern Health Cluster, Dammam, SAU; 4 General Practice, King Saud University, Riyadh, SAU; 5 Otolaryngology, Al-Adwani General Hospital, Ta'if, SAU

**Keywords:** masseteric muscle, pterygoid muscle, soft tissue tumor, maxillofacial region, intramuscular myxoma

## Abstract

Myxomas, characterized by abundant mucoid stroma and spindle cells, represent a subset of benign soft tissue tumors. Intramuscular myxomas in the maxillofacial region are rare, posing diagnostic challenges. We present the case of a 58-year-old male who reported limited jaw movement. Physical examination revealed asymmetry, restricted mouth opening, and left lateral jaw movement. Imaging confirmed a well-defined myxomatous mass. Core needle biopsy confirmed an intramuscular myxoma involving the pterygoid and masseteric muscles. A multidisciplinary team opted for surveillance due to its benign nature. Follow-up at six months showed stable findings, supporting the decision for non-surgical management. This case highlights the diagnostic and management challenges of rare intramuscular myxomas in the maxillofacial region. A comprehensive diagnostic work-up, including clinical, radiological, and histopathological data, is crucial. Non-surgical management, guided by a benign nature, underscores the importance of judicious and multidisciplinary approaches. Regular follow-up contributes to understanding the natural history of intramuscular myxomas, emphasizing the need for vigilant monitoring in soft tissue tumor management.

## Introduction

Myxomas are characterized by the presence of abundant mucoid stroma containing spindle or stellate cells, giving them a distinctive histological appearance. Although often asymptomatic, myxomas can lead to local compression effects, causing pain, swelling, and restricted function in affected areas [1]. Intramuscular myxomas represent a rare subset of benign soft tissue tumors characterized by the proliferation of stellate and spindle-shaped cells embedded within a myxoid matrix. While these lesions can arise in various anatomical locations, their occurrence within the maxillofacial region is exceedingly uncommon [2].

Intramuscular myxomas, specifically those involving the head and neck musculature, remain a diagnostic challenge due to their infrequent presentation and the varied spectrum of clinical manifestations [2]. This case report contributes to the growing body of literature by presenting a detailed clinical account of a 58-year-old male with an intramuscular myxoma involving the left masseteric and pterygoid muscles, resulting in progressively limited jaw movement. This report adds to the sparse literature on intramuscular myxomas by providing a detailed exploration of a rare presentation within the pterygoid and masseter muscles.

## Case presentation

A 58-year-old male patient presented to our outpatient clinic with a chief complaint of limited jaw movement, predominantly on the left side, over the past six months. The patient reported associated discomfort and occasional pain in the left preauricular region. There was no history of trauma, recent dental procedures, or systemic illnesses. His medical history was notable for controlled hypertension and hyperlipidemia, for which he was on regular medications. The patient reported no systemic symptoms such as fever, weight loss, or night sweats. Additionally, he denied any changes in sensation or difficulty with swallowing. Social history did not reveal any significant tobacco or alcohol use.

Physical examination demonstrated a visibly asymmetrical face with limited mouth opening. The maximal inter-incisal distance was measured at 3 cm. Intraoral examination revealed restriction of left lateral jaw movement with palpable resistance. No signs of infection or inflammation were noted.

**Figure 1:**
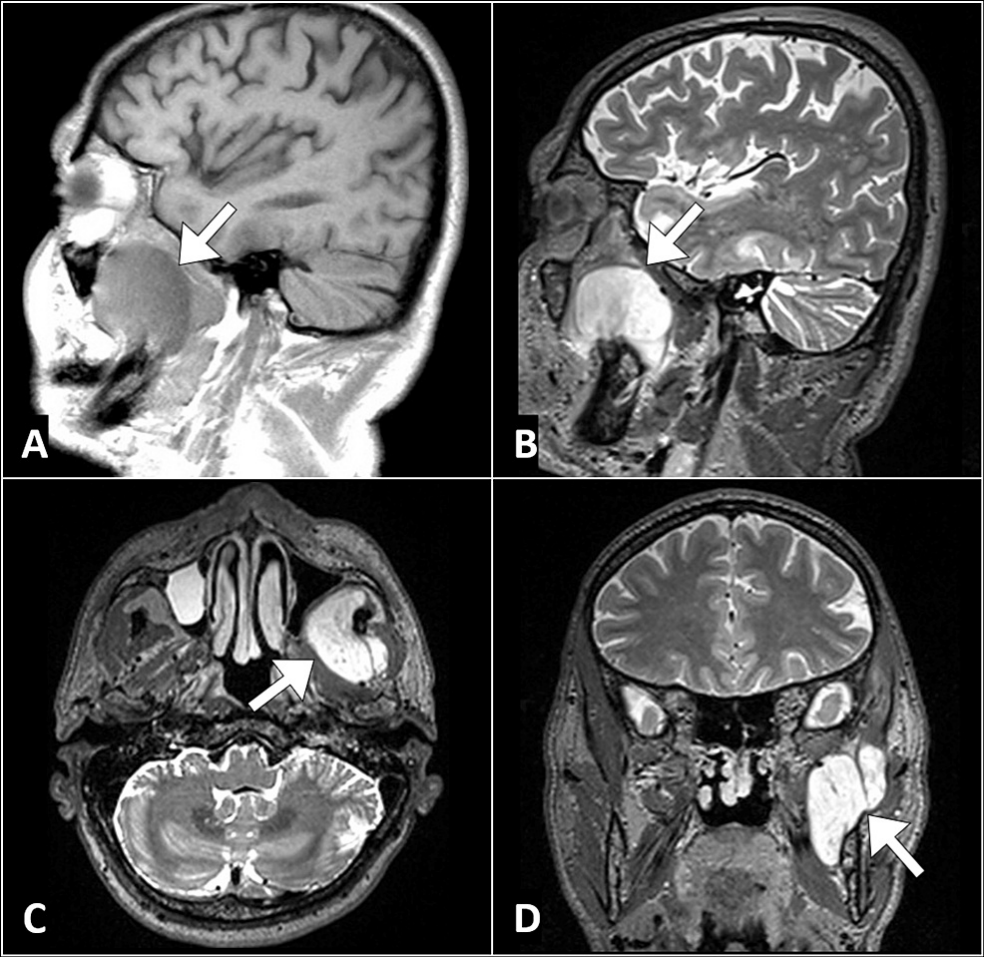
Magnetic resonance images of the brain reveal a lesion in the masseteric muscle (arrow) characterized by low signal intensity on the T1-weighted image (A) and high signal intensity on the T2-weighted image (B). Similar lesions with corresponding intensities were observed in the pterygoid muscles (arrow) as depicted in the axial (C) and coronal (D) T2-weighted images.

Laboratory investigations, including a complete blood count, erythrocyte sedimentation rate, and C-reactive protein, were within normal limits. Imaging studies were then pursued to further evaluate the mass and assess its extent. Magnetic resonance imaging of the head and neck revealed a well-defined intramuscular mass involving the left masseteric and pterygoid muscles. The mass displayed low signal intensity on T1-weighted images and high signal intensity on T2-weighted images, consistent with the characteristics of myxomatous tissue (Figure 1).

**Figure 2:**
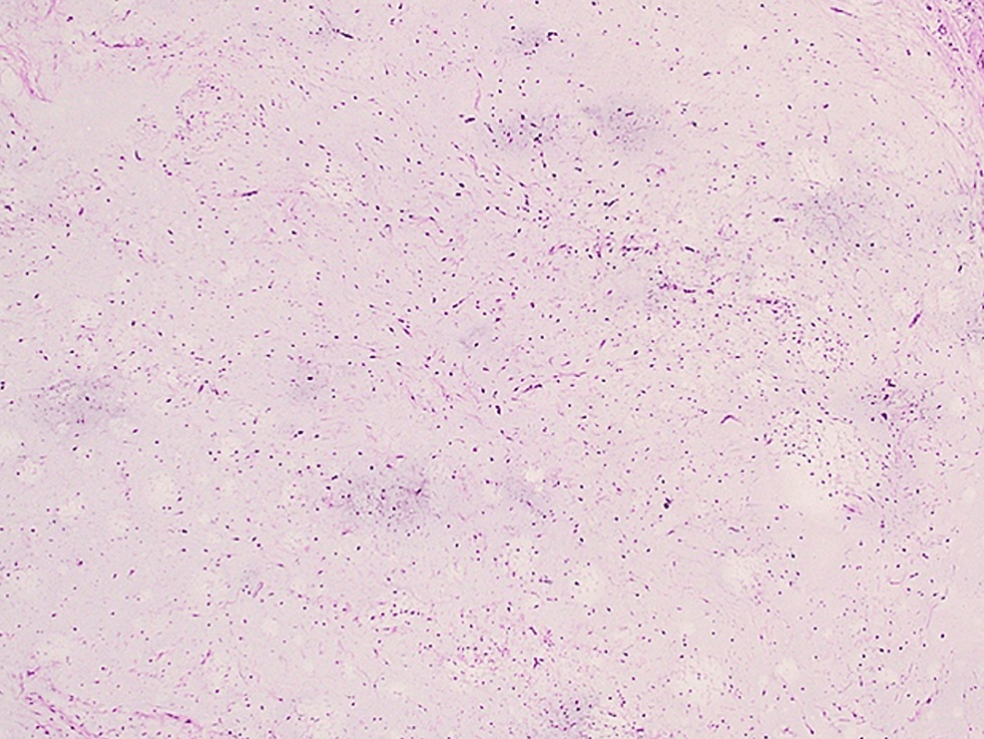
Microscopic examination reveals a proliferation of spindle-shaped cells embedded within a myxoid matrix, confirming the diagnosis of intramuscular myxoma.

Following the imaging studies, a core needle biopsy of the mass was performed. Histopathological examination revealed a proliferation of stellate and spindle-shaped cells embedded in a myxoid matrix, confirming the diagnosis of intramuscular myxoma (Figure 2).

The patient was referred to a multidisciplinary team consisting of oral and maxillofacial surgeons, radiologists, and pathologists. A decision was made to closely monitor the patient rather than pursue surgical excision due to the benign nature of the lesion.

The patient underwent regular follow-up visits in the outpatient clinic to monitor the progression of symptoms and assess the size and characteristics of the myxomatous mass. At the six-month follow-up, the patient reported no worsening in symptoms, and a physical examination revealed almost stable findings. The decision for continued surveillance along with regular sessions of physiotherapy was made, emphasizing the importance of vigilant follow-up in the management of intramuscular myxomas.

## Discussion

The occurrence of an intramuscular myxoma within the pterygoid and masseteric muscles is an extraordinary rarity, as evidenced by the limited reported cases in the literature [2-4]. The deep-seated location of the pterygoid muscle poses diagnostic challenges, as clinical manifestations may be subtle, leading to delayed recognition.

The clinical manifestation of this case primarily centered around gradually progressive limited jaw movement, a symptom that can be attributed to a variety of etiologies, ranging from inflammatory conditions to neoplastic processes. The rarity of intramuscular myxomas in the maxillofacial muscles highlights the need for a high index of suspicion when confronted with similar clinical scenarios [2].

Histopathological examination of the core needle biopsy was instrumental in confirming the diagnosis of intramuscular myxoma. The characteristic proliferation of stellate and spindle-shaped cells within a myxoid matrix is consistent with previous descriptions of this benign neoplasm [5]. The immunohistochemistry results, although omitted in this specific case, are typically positive for vimentin and negative for desmin and S-100 protein, further supporting the diagnosis. The inclusion of these findings in the discussion underscores the importance of integrating clinical, radiological, and histopathological data for a comprehensive diagnostic approach [4,5].

In the absence of malignant features and given the benign nature of intramuscular myxomas, the decision for non-surgical management in this case was made, emphasizing the role of a multidisciplinary team in determining the optimal treatment strategy. This approach aligns with current literature advocating for conservative management when feasible, avoiding unnecessary surgical intervention and potential morbidity associated with resection [3,5].

The reported follow-up, although brief, reflects the stability of the clinical findings of intramuscular myxomas. Regular surveillance is essential to monitor for any changes in symptoms or lesion characteristics. This aligns with the broader consensus in the literature emphasizing the need for vigilant follow-up in the management of soft tissue tumors [4].

## Conclusions

The presented case of intramuscular myxoma involving the left masseteric and pterygoid muscles exemplifies the challenges associated with the diagnosis and management of rare soft tissue neoplasms within the maxillofacial region. The case underscores the critical role of a comprehensive diagnostic work-up, integrating clinical, radiological, and histopathological findings to achieve an accurate diagnosis. The decision for non-surgical management, including regular physiotherapy, highlights the importance of avoiding unnecessary interventions. 
